# Assessment of the efficacy of ChatGPT responses to bacterial species-specific questions in microbiology

**DOI:** 10.1099/acmi.0.001137.v5

**Published:** 2026-08-04

**Authors:** Withanage Dona Manushi Dinasha, Nissanka Mudiyanselage Tanuri Ayanga Nissanka, Chamudhi Prabashi Wickramasinghe, Warnakulasuriya Palakuttige Pasindu Damsara Fernando, Vindya Perera, Hiripitiyage Gayan Danushka Gunatilake

**Affiliations:** 1 Department of Biomedical Science, International Institute of Health Sciences, Welisara, Sri Lanka; 2 Department of Microbiology, Faculty of Medicine, Sabaragamuwa University of Sri Lanka, Belihuloya, Sri Lanka

**Keywords:** AI in education, artificial intelligence, bacterial species, ChatGPT, learning, microbiology

## Abstract

**Background.** ChatGPT, an OpenAI chatbot, serves as a valuable tool in the present for learning and education. It also offers information on microbiology, as its popularity grows among students. However, assessing the accuracy of ChatGPT’s responses is essential due to the potential for ‘hallucinations’ in large language models (LLMs).

**Objectives.** This study focused on evaluating the accuracy of ChatGPT’s responses to general questions on bacterial species and assessing whether the responses included key microbiological terms typically expected in academic or examination settings.

**Methodology.** Questions were designed to reflect interactions at three language proficiency levels, including low, moderate and high. A clinical microbiologist finalized a list of 15 bacterial species, each with 18 specific questions of both local and international relevance. These questions were then prompted to ChatGPT 3.5 and 4.1 mini models, simulating real user interactions. Responses were evaluated using a microbiology reference guide and categorized as accurate, mixed/incomplete or inaccurate.

**Results.** Results revealed average scores of 1.5%, 58.1% and 40.4% and 0.5%, 43.2% and 56.3% for inaccurate, mixed/incomplete and accurate answers for 3.5 and 4.1 mini models, respectively. While high proficiency demonstrated a higher percentage of accurate responses, all other results were either mixed/incomplete or inaccurate.

**Conclusion.** Findings suggest that precise questions yielded more accurate responses, while imprecise questions often led to partially correct responses. Notably, ChatGPT 4.1 mini gave clearer and more reliable answers than ChatGPT 3.5. The study emphasizes the influence of question formulation on response accuracy, recommending further research to explore more advanced LLMs like ChatGPT-4o and ChatGPT-o3 models.

**Impact Statement**

This study provides a novel evaluation of ChatGPT’s accuracy and content quality across three proficiency levels in response to microbiology-related queries, using outputs from versions 3.5 and 4.1 mini. By applying a keyword-based scoring framework rooted in a clinical microbiology reference guide, it reveals how artificial intelligence (AI)-generated answers vary with question specificity and complexity. This adds to existing literature by introducing a structured, proficiency-based benchmarking approach relevant to education in microbiology. The findings have broad utility, especially in academic settings, where students must meet the expectations in assessments. The study highlights how ChatGPT’s outputs can expose the reliance on specific keywords in exam scoring, an insight valuable for learners. It emphasizes that, beyond factual accuracy, exam success often hinges on using the key terms, which AI models may or may not generate depending on their proficiency level. Importantly, this work demonstrates ChatGPT’s potential as a reflective tool for assessment readiness, helping students recognize gaps in their exam-oriented language. By aligning AI performance with academic evaluation patterns, this study reinforces the value of AI not just for learning content but for improving alignment with how knowledge is tested.

## Data Summary

The authors confirm that all supporting data, code and protocols have been provided within the article or through supplementary data files.

## Introduction

Bacteria, among the most diverse and ubiquitous micro-organisms on Earth, play critical roles in ecosystems, industry, medicine and human health. The discovery of numerous new bacterial species globally in recent years has underscored their diverse impacts [1, 2]. While many bacteria contribute to advancements in bioremediation [3] and the understanding of the human microbiome [4], others have emerged as significant threats, causing infectious disease outbreaks and straining healthcare systems worldwide [5]. According to the World Health Organization [6], the 2024 Bacterial Priority Pathogens List emphasizes the global threat posed by antibiotic-resistant pathogens, categorizing 15 families into critical, high and medium priorities. Critical-priority pathogens, such as *Acinetobacter baumannii*, rifampicin-resistant *Mycobacterium tuberculosis* and Enterobacterales, represent severe risks, particularly in low- and middle-income countries. High-priority pathogens include *Salmonella*, *Shigella*, *Pseudomonas aeruginosa*, *Staphylococcus aureus* and *Neisseria gonorrhoeae*, while medium-priority pathogens, such as *Streptococcus* and *Haemophilus influenzae*, disproportionately impact vulnerable populations in resource-limited settings [7]. Given their significant impact, understanding bacterial species, their characteristics and their behaviour is fundamental in fields like microbiology, biotechnology and clinical research [1].

In spite of the importance of microbiology, many students struggle with retaining and applying bacterial knowledge due to the sheer volume of information, complexity of bacterial classification and rapid advancements in microbial research [8]. Traditional teaching methods, while effective, are often supplemented with digital tools and online resources to enhance student engagement and learning outcomes [9]. In recent years, the emergence of large language models (LLMs) has transformed access to knowledge across various disciplines, including modern research into bacterial pathogenesis, antimicrobial resistance and diagnostic advancements [10, 11]. LLMs are advanced artificial intelligence (AI) systems capable of processing and generating human-like text based on extensive training datasets [10, 12, 13]. Among these, ChatGPT (Chat-Generative Pre-Trained Transformer) [14] gained significant popularity among students and educators due to its ease of access, versatility and ability to provide instant responses to queries [15, 16]. It reached one million users just 5 days after its launch and surpassed 100 million users in less than 2 months [17]. By leveraging their capabilities, ChatGPT can play a transformative role in educating people about infectious bacterial diseases, enhancing understanding and fostering proactive behaviours to reduce their impact globally [18].

However, while ChatGPT is widely used, its outputs are not consistently verified for accuracy, especially in specialized fields such as microbiology [19]. A well-documented limitation of LLMs, including ChatGPT, is the phenomenon of 'hallucinations', instances where the model generates plausible-sounding but incorrect or nonsensical information [20, 21]. This raises concerns about the reliability of ChatGPT as a tool for academic and scientific learning [22]. While studies have evaluated ChatGPT’s accuracy within the fields of medical and healthcare education [23, 24], limited research has specifically assessed its accuracy in microbiology or examined its academic utility within the Sri Lankan higher education context. The primary objective of this study is to systematically assess the accuracy and efficacy of ChatGPT responses to bacterial species-specific questions in microbiology. By evaluating the correctness, consistency and reliability of these responses, this research aims to provide insights into the potential and limitations of using ChatGPT as a supplementary educational resource in microbiology. In order to align with real-world academic assessment practices, this study not only evaluates the factual accuracy of AI-generated answers but also highlights the importance of keyword usage in structured assessments where marks are awarded based on specific terminology. Therefore, this study aimed to evaluate the accuracy of responses generated by the ChatGPT AI language model, specifically utilizing its GPT-3.5 and 4.1 mini versions.

## Methods

To initiate the process, a comprehensive list of bacterial species and relevant questions was developed in collaboration with a board of clinical microbiologists. The selected bacterial species were chosen based on their national importance in seasonal epidemics of Sri Lanka, as reported by the national health ministry [25, 26] and their common presence in medical and educational curricula. This selection of a total number of 15 bacterial species ensured the study’s relevance to both clinical practice and academic learning environments. Consequently, a total of 18 standardized question types were developed, and each of these questions was reformulated into 3 proficiency-level versions, including low, moderate and high, to reflect varying levels of linguistic and technical complexity. Accordingly, for each of the 15 selected bacterial species, 3 sets of 18 questions (1 per proficiency level) were applied, resulting in a total of 810 (18 questions×15 species×3 proficiency levels) individual prompts per ChatGPT model (see File S1). In this study, technical terminology is defined as domain-specific microbiological terms commonly used in academic and clinical contexts, including those related to bacterial identification (i.e. Gram staining and culture characteristics), diagnostic methods (i.e. biochemical tests and polymerase chain reaction) and pathogenic features (i.e. virulence factors). At the low proficiency level, the questions were simple, using up to one to two technical terminologies and framed in a general, non-specific manner to test the model’s capability with basic queries (i.e. how do you identify *Streptococcus*?) Whereas with moderate proficiency, the questions were moderately technical up to three to four terminologies and often incomplete, requiring the model to gather missing details and deliver logical answers (i.e. how to identify *Streptococcus pyogenes*?). Hence, at the high proficiency level, the questions incorporated advanced technical terms up to five to six, highly specific, precise and contextually accurate questions to assess the model’s ability to handle complex and detailed inquiries (i.e. what are the microbial diagnostic methods of *S. pyogenes* in a laboratory?) (File S2 – Table S1, available in the online Supplementary Material).

Each level of proficiency was triplicated on the GPT-3.5 and 4.1 mini versions to ensure a robust and systematic approach to data collection. Hence, the triplication was performed by independently replicating each proficiency level three times using three different users (User 1, User 2 and User 3) operating separate devices, minimizing potential user-specific or device-related biases. This replication was performed for all 18 standardized questions across 15 bacterial species, resulting in a total of 810 responses analysed per ChatGPT version. These versions were chosen primarily because it is freely available to the public, ensuring accessibility and reproducibility of the study by other researchers [17, 27]. Although GPT-4o is available, its free usage is restricted [28]. Therefore, GPT-3.5 and GPT-4.1 mini were selected as the most practical and consistent models for this study. Responses were collected in February 2024 for GPT-3.5 and in April 2025 for GPT-4.1 mini.

During the data collection phase, the finalized questions were input into ChatGPT under controlled and consistent conditions, which were the utilizing of the same version, the same prompting time and date and the same order of the questions. For each user, all questions were entered sequentially within a single session per model, rather than initiating a new session for each query. Additionally, the ‘regenerate response’ function was not utilized, and only the initial response generated by the model was considered for analysis to ensure consistency across all trials. After that, the data analysis process consisted of both quantitative and qualitative components. The qualitative assessment considered correctness, clarity and contextual relevance of responses. Correctness was evaluated using the 18th edition of the Medical Microbiology Guide [29], which served as the reference standard. Clarity and contextual relevance were assessed based on whether the responses were appropriately structured and directly addressed the question. The final classifications were validated by a clinical microbiologist to ensure alignment between keyword-based scores and overall microbiological accuracy.

Quantitative analysis assessed response completeness using a keyword-based scoring system. This scoring was used to generate the initial classification of responses, which was subsequently interpreted and validated through qualitative analysis. This dual-method approach, combined with structured question categorization and multi-device validation, ensured a comprehensive and reliable evaluation of GPT-3.5 and 4.1 mini accuracy in responding to microbiology-related queries.

Hence, for each question, key microbiological terms were identified based on the Medical Microbiology Guide (Greenwood *et al*., 18th edition). These keywords were defined as essential diagnostic, structural or functional descriptors expected in an academically correct answer. The initial keyword list was developed by the research team and subsequently reviewed and validated by a qualified clinical microbiologist (affiliated with an academic institution) to ensure accuracy and relevance. The validation involved assessing whether the selected keywords and response classifications appropriately reflected correct and contextually relevant microbiological content. The microbiologist was not involved in the initial scoring process, and validation was conducted as part of the overall study framework.

**Fig. 1.**
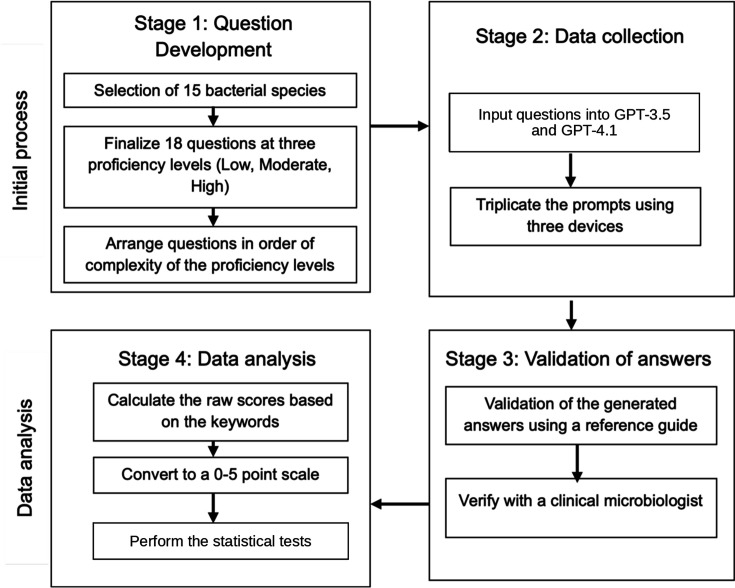
Flowchart illustrating the methodological framework of the study.

The total number of expected keywords varied depending on the question. Each response was analysed to determine how many expected keywords were correctly included. The raw score defined as the number of matched keywords was then converted into a 0–5-point scale (see supplementary files – Excel file). For example, if the expected number of keywords was 12 and a response contained 5 correct keywords, the proportion (5 out of 12) was calculated and mapped to a value on the 0–5 scale. The conversion was applied consistently across all responses to ensure objective comparison. Responses scoring 0 were considered inaccurate, those scoring between 1 and <5 were classified as mixed or partially complete, and responses scoring exactly 5 were classified as fully accurate. The average of three scores of each user in each proficiency level was taken as the final score for analysis with the use of non-parametric and descriptive statistical tests. Thereafter, these categorized responses were verified by clinical microbiologists to confirm that the scoring and classification accurately reflected the content quality, thereby ensuring the integrity and accuracy of the final qualitative analysis (Fig. 1).

## Results

### Overall performance of ChatGPT models across users and proficiency levels

A total of 810 responses were evaluated for each version of ChatGPT (3.5 and 4.1 mini) across 3 systematically designed prompts. These prompts were structured to reflect increasing levels of user proficiency, including low, moderate and high.

Table 1 presents the descriptive statistics, including the mean and standard deviation for each prompt and the overall average. ChatGPT 4.1 mini demonstrated superior performance across all prompts, with an overall mean score of 4.216 compared to 3.794 for ChatGPT 3.5. This indicates that ChatGPT 4.1 mini generally provided more accurate and complete responses. The lower standard deviation for ChatGPT 4.1 mini (1.09) relative to ChatGPT 3.5 (1.28) suggests greater consistency in response quality.

**Table 1. T1:** Descriptive statistics and proficiency-level performance of ChatGPT 3.5 and ChatGPT 4.1 mini

	Parameter	ChatGPT 3.5	ChatGPT 4.1 mini
**Descriptive performance across users**	User 1 (mean±sd)	3.80±1.28	4.21±1.12
	User 2 (mean±sd)	3.78±1.28	4.22±1.11
	User 3 (mean±sd)	3.80±1.29	4.21±1.12
	**Overall average (mean±****sd****)**	**3.79±1.23**	**4.22±1.09**
**Model performance across proficiency levels**	Low proficiency (mean±sd)	3.08±1.37	3.75±1.27
	Moderate proficiency (mean±sd)	3.83±1.04	4.24±1.01
	High proficiency (mean±sd)	4.46±0.81	4.65±0.72

The results show that ChatGPT 4.1 mini consistently achieved higher mean scores with lower standard deviations compared to ChatGPT 3.5, indicating more accurate and consistent performance. Performance increased progressively from low to high proficiency for both models, demonstrating that question specificity and technical detail significantly improved response accuracy. The highest mean scores were observed at the high proficiency level, particularly for ChatGPT 4.1 mini.

Interestingly, both models achieved the maximum score of 5.00 during the prompting by all three users (User 1, User 2 and User 3), indicating that the highest performance was observed across all user prompts yet also recorded a minimum score of 0.00, revealing that both versions occasionally generated completely inaccurate responses.

The median and mode values for ChatGPT 4.1 mini were uniformly 5.00 across all prompts, further supporting the interpretation that this version delivered more consistently high-scoring responses. These values provide a quantitative foundation for subsequent analyses, highlighting the consistent advantage of GPT-4.1 mini in delivering high-quality responses across various question complexities (Table 2).

**Table 2.** Performance evaluation of GPT-3.5 and ChatGPT 4.1 mini across graded microbiology prompts

Model	Prompt	Observed response	Performance classification
**ChatGPT 3.5**	Is *Escherichia coli* Gram positive or negative?	*Escherichia coli* (*E. coli*) is a Gram-negative bacterium. Gram-negative bacteria like *E. coli* have a cell wall structure that includes a thin layer of peptidoglycan surrounded by an outer membrane. When subjected to the Gram staining technique, Gram-negative bacteria appear pink or red under a microscope.	Accurate and contextually appropriate
**ChatGPT 4.1 mini**	Is *Escherichia coli* Gram positive or negative?	*Escherichia coli* is classified as a Gram-negative bacterium because it cannot retain the crystal violet stain during Gram staining due to its thin peptidoglycan layer and outer lipid membrane. During the alcohol decolorization step, the crystal violet-iodine complex is washed out, and the bacterium then takes up the safranin counterstain, appearing pink or red under the microscope. This pink/red appearance identifies *E. coli* as Gram-negative.	Accurate and comprehensive

The table demonstrates the performance of GPT-3.5 and ChatGPT 4.1 mini across high-proficiency microbiology questions and illustrates differences in response depth, contextual relevance and completeness between the models. ‘Accurate and contextually appropriate’ describes responses that were correct and relevant but concise, whereas ‘accurate and comprehensive’ refers to responses that were correct, detailed and included broader explanatory information.

**Fig. 2.**
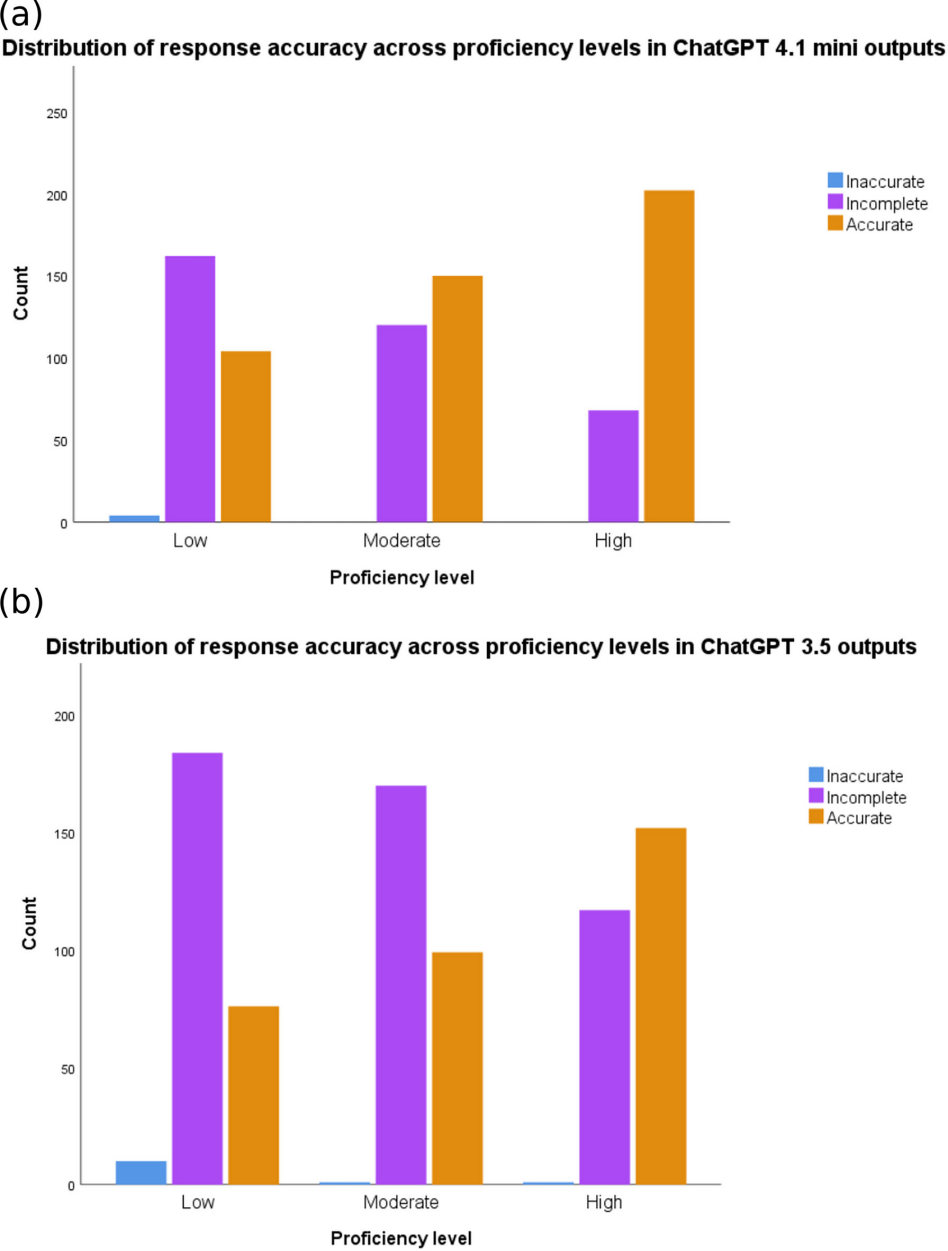
The distribution of response accuracy across proficiency levels in 4.1 mini and ChatGPT 3.5 outputs. The following figure compares the distribution of response accuracy (inaccurate, incomplete and accurate) across low, moderate and high proficiency question categories for ChatGPT 4.1 mini (panel a) and ChatGPT 3.5 (panel b).

**Fig. 3.**
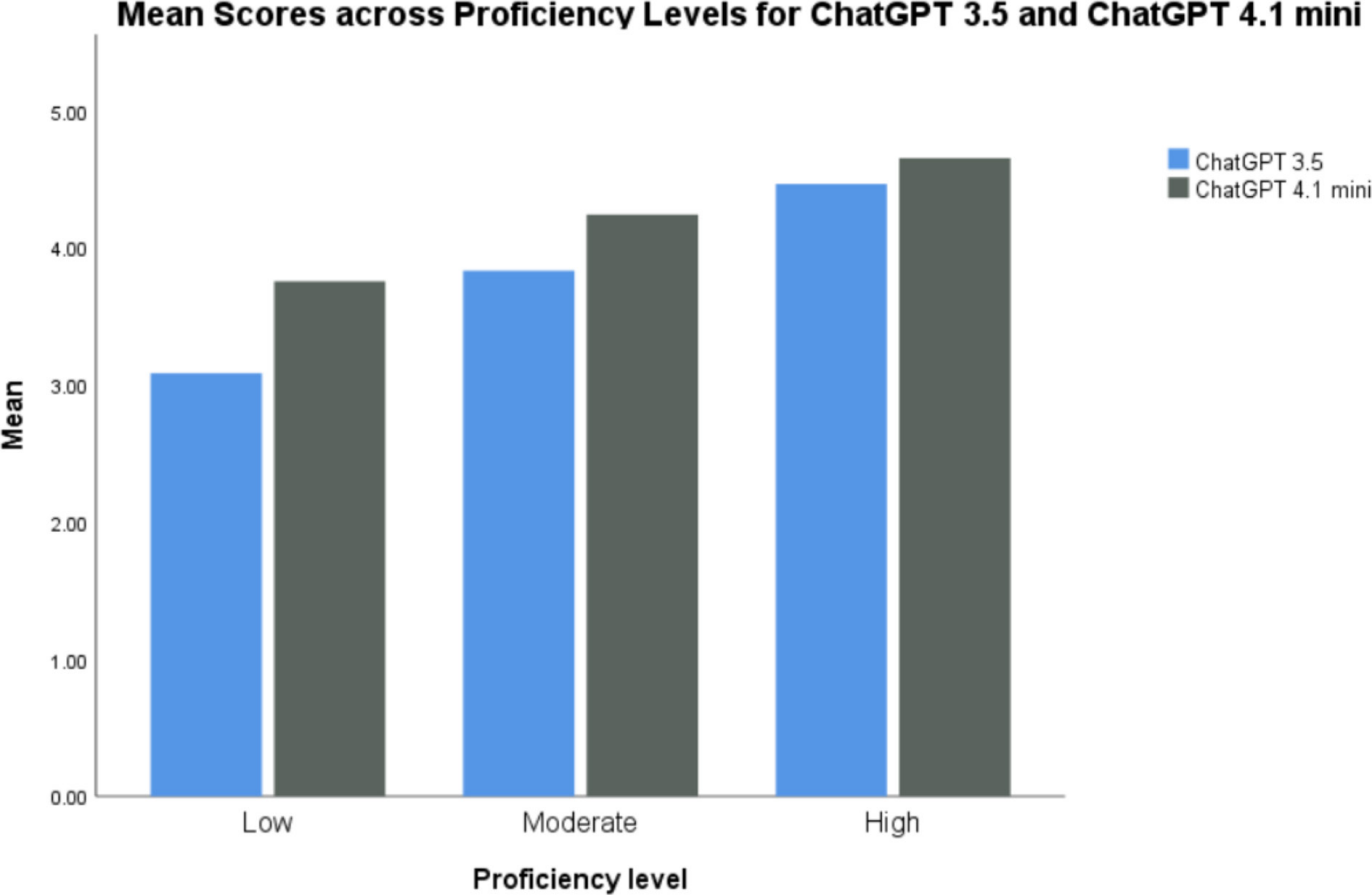
The average mean scores of ChatGPT 3.5 and ChatGPT 4.1 mini across three proficiency levels. The bar graph compares the average mean scores of ChatGPT 3.5 and ChatGPT 4.1 mini across three proficiency levels, including low, moderate and high. Across all levels, ChatGPT 4.1 mini consistently outperforms ChatGPT 3.5. The performance gap is most notable at the low proficiency level, where ChatGPT 4.1 mini scores significantly higher than version 3.5. At the high proficiency level, both models perform similarly; however, ChatGPT 4.1 mini still shows a slight edge, reflecting its ability to maintain strong performance even with complex or advanced queries. This trend suggests that ChatGPT 4.1 mini provides a more robust and consistent response quality across users with varying language proficiency.

Both models demonstrated improved accuracy with increasing proficiency level (see File S2 – Table S1). This indicates that the models performed better when prompted with more specific, detailed and technically complex questions. Both models achieved their highest scores when presented with highly technical, well-structured prompts in high proficiency, while low average scores were in the low proficiency level (Fig. 2). For ChatGPT 3.5, mean scores increased from 3.08 (low proficiency) to 4.46 (high proficiency). Similarly, ChatGPT 4.1 mini-improved from 3.75 (low) to 4.65 (high) (Fig. 3 and Table 1), showing particularly strong performance when responding to technically detailed and well-structured prompts. These patterns highlight a consistent trend in which higher proficiency questions elicited more accurate, keyword-rich responses from both models.

### Normality testing of ChatGPT scores across prompts and proficiency level

All prompts for both ChatGPT 3.5 and ChatGPT 4.1 mini yielded *P*-values less than 0.001, confirming statistically significant deviations from normality in all cases (Table 3). This suggests that the distribution of scores is irregular and inconsistent with the assumptions of normality.

**Table 3.** Statistical comparison of ChatGPT 3.5 and ChatGPT 4.1 mini across prompts and proficiency levels

	Statistic	ChatGPT 3.5	ChatGPT 4.1 mini
**Friedman test (prompt variation)**	Sample size (N)	810	810
	Chi-square (χ²)	2.489	0.041
	df	2	2
	***P*-value**	**0.288**	**0.980**
**Kruskal–Wallis test (proficiency level differences)**	Chi-square (χ²)	151.519	90.097
	Mean rank – low proficiency	284.08	319.38
	Mean rank – high proficiency	523.36	492.54
	df	2	2
	***P*-value**	**<0.001**	**<0.001**

The Friedman test showed no statistically significant variation (*P*>0.05) across the three replicated user prompts, indicating consistent model performance regardless of the user or device. In contrast, the Kruskal–Wallis test revealed highly significant differences across proficiency levels for both models (*P*<0.001), with mean rank scores increasing from low to high proficiency. These results confirm that technical complexity and structured question design strongly influence the accuracy of ChatGPT-generated microbiology responses.

Further average scores of both models at each proficiency level (low, moderate and high) also demonstrated highly significant non-normal distributions (*P*<0.001 for all comparisons). These consistent findings across both prompt-level and proficiency-level groupings validate the non-normal structure of the score data in this study.

Such a distributional pattern may reflect the variation in model responses, the influence of question complexity and scoring extremes (e.g. accurate, mixed or inaccurate). These results consistently demonstrate that the score distributions of both ChatGPT versions deviate significantly from normality at both prompt and proficiency levels.

### Average score comparison of prompt and proficiency levels for model evaluation

Comparing the three prompts in ChatGPT version 3.5 and version 4.1, the Friedman test gives the *P*-value of 0.288 and 0.980 (Table 3), suggesting that there is no statistically significant difference between these three prompts at 5% level of significance for both versions. According to the Kruskal–Wallis test for both models, low proficiency level obtained a low mean rank (284.08 for ChatGPT 3.5 and 319.38 for ChatGPT 4.1 mini), while the high proficiency level obtained a high mean rank (523.36 for ChatGPT 3.5 and 492.54 for ChatGPT 4.1 mini). This indicates that questions framed with more technical terminologies that are highly precise and specific lead both models to generate more accurate answers. The *P-*values show (*P*<0.001) highly statistically significant differences among the three proficiency levels for both models. Furthermore, these results indicate that user proficiency level has a substantial impact on the accuracy and quality of responses generated by both ChatGPT versions.

### Performance comparison between ChatGPT 3.5 and 4.1 mini models

A statistical comparison of the average scores between the two versions (Table 3) revealed a highly significant difference, with ChatGPT 4.1 mini (Z=−10.2, *P*<0.001) demonstrating notably better performance than ChatGPT 3.5, highlighting a marked improvement in response accuracy and quality, reflecting the ongoing developmental progress of OpenAI’s ChatGPT models since their initial release. Table 4 defines the distribution of average scores across defined ranges in ChatGPT 3.5 and 4.1. This further supports ChatGPT 4.1 mini for educational support in microbiology contexts.

**Table 4.** The distribution of average scores across defined ranges in ChatGPT 3.5 and ChatGPT 4.1 mini models

Score range	ChatGPT 3.5 (%)	ChatGPT 4.1 mini (%)
0–1	1.98%	1.11%
1–2	7.16%	4.07%
2–3	21.11%	12.10%
3–4	21.98%	17.90%
4–5	47.78%	64.81%

The table compares the distribution of average response scores between ChatGPT 3.5 and ChatGPT 4.1 mini.

## Discussion

### Comparative analysis of ChatGPT 3.5 and GPT-4.1 mini performance

The comparative analysis between ChatGPT version 3.5 and GPT-4.1 mini revealed that while both models performed well in answering bacterial species-specific microbiology questions, GPT-4.1 mini demonstrated more consistent and reliable performance across all proficiency levels. Both models performed well overall, with average scores above 3.5 out of 5. However, GPT-4.1 mini exhibited lower variability in responses, indicating a more stable and predictable output quality. Both versions showed an upward trend in performance from low to high proficiency questions, but GPT-4.1 mini displayed a smoother and more linear progression, suggesting improved handling of complex queries. In both models, a progressive improvement in performance is observed as question proficiency increases. Specifically, accurate responses rise steadily from low to high proficiency levels, while the frequency of incomplete and inaccurate outputs declines. This upward performance gradient is more evident in ChatGPT 4.1 mini. At the low proficiency level, both models generate a substantial proportion of incomplete responses. However, ChatGPT 4.1 mini demonstrates a comparatively lower occurrence of inaccurate outputs (Fig. 2). As proficiency increases, ChatGPT 4.1 mini exhibits a marked shift towards accurate responses, particularly at the high proficiency level, where accurate outputs clearly predominate and incomplete responses are substantially reduced. In contrast, although ChatGPT 3.5 also shows improved accuracy with increasing proficiency, it retains a relatively higher proportion of incomplete responses across all levels, including the high proficiency category (Fig. 2 and Table 4). This suggests comparatively greater response fragmentation and reduced content completeness in the earlier model. These findings align with previous investigations comparing GPT-3.5 and GPT-4 in medical education contexts, where GPT-4 consistently achieved superior accuracy, stronger reasoning ability and improved performance on complex medical examinations compared with GPT-3.5, indicating enhanced capability in handling specialized biomedical knowledge [30]. Overall, the distributional pattern demonstrated greater precision, completeness and consistency in responses generated by ChatGPT 4.1 mini, particularly for complex and specialized microbiology questions. While these findings suggest a positive relationship between question specificity and response quality, previous studies have indicated that LLM outputs may be influenced by conversational context and sequential prompting, where information from earlier interactions contributes to improved performance in subsequent responses [31]. Therefore, the possibility that contextual memory effects influenced later responses within the same session should also be considered. Consequently, improvements observed across proficiency levels may partly reflect contextual carryover effects in addition to the intrinsic performance of the model.

In terms of score distribution (Table 4), both models had ~50% of their responses rated at 4 or 5. Therefore, in the ChatGPT 3.5 model, nearly half of the responses (47.78%) fell within the 4–5 range, indicating a substantial proportion of accurate or near-accurate answers. Moderate performance was also notable, with 21.98 and 21.11% of responses in the 3–4 and 2–3 ranges, respectively. Lower scores were less frequent, with 7.16% in the 1–2 range and 1.98% in the 0–1 range, reflecting a smaller proportion of incomplete or incorrect responses. In contrast, ChatGPT 4.1 mini demonstrated a clear improvement in score distribution, with a markedly higher proportion of responses (64.81%) in the 4–5 range. The percentages in the 3–4 (17.50%) and 2–3 (12.10%) categories were lower than those observed for ChatGPT 3.5, while low-scoring responses were comparatively rare at 4.07% [1, 2] and 1.11% (0–1). Overall, these findings indicate that ChatGPT 4.1 mini produced more consistently high-quality responses with fewer instances of moderate or low accuracy compared to ChatGPT 3.5.

Moreover, statistical tests confirmed that GPT-4.1 mini had clearer group separation and more robust differentiation across proficiency levels ([Table T1]). This was further supported by narrower standard deviations and tighter confidence intervals in the high proficiency group, reflecting greater consistency in the quality of responses. Overall, while ChatGPT 3.5 demonstrated notable capabilities in responding to microbiology-related queries, the GPT-4.1 mini model consistently outperformed it in terms of accuracy, reliability and adaptability, particularly as the technical complexity of questions increased. These findings suggest that GPT-4.1 mini holds greater potential for use in academic microbiology education as a supporter tool, where precision and contextual accuracy are essential. Similar trends have been reported in broader healthcare and educational research evaluating newer generations of LLMs. Previous studies comparing GPT-3.5 and GPT-4-based models in medical education and certification assessments have demonstrated improvements in factual accuracy, contextual reasoning and response consistency in the newer models [30, 32]. The marked improvement between the two versions underscores the effect of OpenAI’s continuous improvement approach. Over the years, ongoing refinements in the architecture and training of these models have translated into progressively better performance, enabling GPT-4.1 mini to respond more effectively to domain-specific, high-proficiency questions. This evolution highlights the growing utility of AI models as supplementary tools in knowledge-intensive fields.

### Accuracy and reliability of ChatGPT in microbiology education

The findings of this study highlight that ChatGPT provides generally accurate responses to bacterial species-specific microbiology questions, particularly at moderate and high proficiency levels. The mean scores across all responses were recorded as 4.46 for 3.5 and 4.65 for 4.1 mini models in the high proficiency group suggesting that the technical term quality is significant for the output and that prompt construction and technical specificity had a greater influence on response quality than session history effects (Fig. 3 and Table 1). These results align with previous studies evaluating ChatGPT’s performance in medical and healthcare education, which found that the model demonstrates competency in factual recall but may struggle with nuanced or highly specialized questions [23, 24]. Studies by [23] and [33] have reported similar findings, noting that while ChatGPT is effective in answering medical board-style questions, it occasionally lacks depth in its explanations [28]. The higher accuracy at high proficiency levels suggests that ChatGPT performs best when responding to well-structured, detailed queries, a finding consistent with research on LLMs in science education [10, 13]. However, variability in ratings across proficiency groups indicates that the model’s performance may be influenced by user expertise, as more knowledgeable individuals may better interpret and assess AI-generated responses.

### Limitations in contextual understanding and technical precision

Despite ChatGPT’s strong performance in general microbiology knowledge, limitations were evident in handling complex or highly technical questions. The presence of lower ratings, particularly at the low proficiency level, suggests that ChatGPT occasionally generates responses lacking precise contextual understanding or depth. This aligns with prior research that highlights the phenomenon of 'hallucinations' in LLMs, where AI models produce plausible yet incorrect information [20, 21]. A study [34] on the use of LLMs in scientific writing also identified issues with factual consistency, indicating that AI-generated content often requires human verification. Additionally, the lack of real-time access to updated scientific literature may contribute to inconsistencies in responses, particularly for bacterial species included in this study that are associated with evolving antimicrobial resistance patterns and continuously updated clinical knowledge, such as *S. aureus* and *N. gonorrhoeae* [19, 22]. This may have contributed to some variability observed in the generated responses. These challenges reinforce the importance of supplementing AI-generated content with verified academic sources and expert validation in microbiology education.

Previous evidence suggests that LLMs can use information from earlier prompts within the same conversation to shape later responses, which may improve coherence, consistency and perceived accuracy over time [31]. Therefore, responses produced at higher proficiency levels may have been influenced, to some extent, by contextual information carried over from earlier interactions rather than reflecting only the model’s independent ability to answer each question.

### Implications for educational use and student engagement

The results indicate that ChatGPT can serve as a valuable supplementary educational tool for microbiology students, particularly in reinforcing foundational concepts and enhancing engagement with microbiological topics. Studies on AI integration in education have shown that LLMs can improve student interaction and comprehension, particularly when used alongside traditional teaching methods [9, 10]. Furthermore, studies by [35, 36] demonstrated that AI-based learning assistants improved student engagement in medical sciences by providing interactive explanations and immediate feedback. However, given the inconsistencies identified in this study, ChatGPT should not be solely relied upon as an authoritative source. Instead, it should be integrated with expert-reviewed materials to ensure accuracy and credibility. Moreover, educators should encourage critical thinking and verification skills among students using AI-assisted learning tools to minimize the risks associated with misinformation.

### Future directions and recommendations

While this study provides valuable insights into the efficacy of ChatGPT in responding to bacterial species-specific microbiology questions, further research is needed to enhance its application in scientific education. Future studies could focus on evaluating GPT-4o or later versions, given their potential improvements in accuracy and contextual reasoning. Research by [37–39] has already suggested that GPT-4o exhibits a more refined understanding of medical and microbiology terminology and reasoning compared to its predecessors. Additionally, investigating ChatGPT’s effectiveness in specialized microbiology subfields, such as antimicrobial resistance mechanisms or molecular pathogenesis, could provide deeper insights into its strengths and limitations. Developing AI models with real-time literature access and expert-verified datasets could further enhance their reliability as educational tools.

## Supplementary material

10.1099/acmi.0.001137.v5 Supplementary Material 1.

10.1099/acmi.0.001137.v5 Supplementary Material 2.

10.1099/acmi.0.001137.v5 Supplementary Material 3.

## Abbreviations

AI: artificial intelligence
ChatGPT: Chat-Generative Pre-Trained Transformer
LLMs: large language models
