# Supplementary material for: Assessment of the efficacy of ChatGPT responses to bacterial species-specific questions in microbiology
Source: Access Microbiol. 2026 Aug 4;8(8):001137.v5. doi: 10.1099/acmi.0.001137.v5 (PMC13449012; doi:10.1099/acmi.0.001137.v5)
Supplement: Supplementary file 1 [file acmi-8-01137-s002.pdf]

Question list- *Staphylococcus aureus*

Low proficiency

1. Is *Staphylococcus* positive or negative?
2. What family does *Staphylococcus* fall under?
3. What is the shape of *S. aureus*?
4. What is the shape of the growing bacterial culture of *S. aureus*?
5. What are the chemical characteristics of *Staphylococcus*?
6. How do you identify *Staphylococcus*?
7. Where does *Staphylococcus* usually live?
8. What are the infections caused by *Staphylococcus*?
9. What are the diseases caused by *Staphylococcus*?
10. What are the sample collection methods for the diseases caused by *Staphylococcus*?
11. How does *Staphylococcus* spread?
12. What are the media used to grow *Staphylococcus*?
13. At which temperature does *Staphylococcus* grow?
14. What is the appearance of *Staphylococcus* in the media?
15. Does *Staphylococcus* form spores?
16. What are the media used to isolate *Staphylococcus*?
17. What are the antibiotics used for *Staphylococcus*?
18. What is the response to the antibiotics used for *Staphylococcus*?

Moderate proficiency

1. Is *S. aureus* gram-positive or negative?
2. What family does *S. aureus* fall under?
3. What is the physical shape of *Staphylococcus aureus*?
4. What is the shape of growing *Staphylococcus aureus* colonies in agar?
5. What are the biochemical characteristics of *S. aureus*?
6. How to identify *S. aureus*?
7. Where does *S. aureus* live?

8. What are the hospital-acquired infections caused by *S. aureus*?
9. What are the identified diseases caused by *S. aureus*?
10. What are the sample collection methods for the diseases caused by *S. aureus*?
11. How does *S. aureus* transmit?
12. What are the culture media used to grow *S. aureus*?
13. What are the temperatures of growing *S. aureus*?
14. What is the appearance of *S. aureus* in culture media?
15. Does *S. aureus* form spores?
16. What are media used to isolate *S. aureus*?
17. What are the antibiotics used for *S. aureus*?
18. What is the bacterial response to the antibiotics used for *S. aureus*?

High proficiency

1. Is *Staphylococcus aureus* gram-positive or negative?
2. What family does *Staphylococcus aureus* fall under the classification of microorganisms?
3. What is the physical shape of the live cultured *Staphylococcus aureus*?
4. What are the colony characteristics of *Staphylococcus aureus* in nutrient agar?
5. What are the biochemical characteristics of *Staphylococcus aureus*?
6. What are the microbial diagnostic methods of *Staphylococcus aureus* in a laboratory?
7. What are the known habitats of *Staphylococcus aureus*?
8. What are the community-acquired and hospital-acquired infections caused by *Staphylococcus aureus*?
9. What are the identified diseases caused by *Staphylococcus aureus*?
10. Specific sample collection methods for the diseases caused by *Staphylococcus aureus*?
11. What are the transmission methods of *Staphylococcus aureus*?
12. What are the specific culture media used to grow *Staphylococcus aureus*?

13. What are the general temperatures of growing *Staphylococcus aureus*?
14. What is the morphological appearance of *Staphylococcus aureus* in specific culture media?
15. Does *Staphylococcus aureus* form spores?
16. What are the specific media used to isolate *Staphylococcus aureus*?
17. What is the antibiogram of *Staphylococcus aureus*?
18. What is antibiotic resistance for *Staphylococcus aureus*?

Note: The following example set of 18 questions (across three proficiency levels) was used for all 15 bacterial species (which are provided below), with only the species name substituted accordingly.

Bacteria list

1. *Staphylococcus aureus*
2. *Streptococcus pyogenes*
3. *Vibrio cholerae*
4. *Mycobacterium leprae*
5. *Helicobacter pylori*
6. *Neisseria gonorrhoeae*
7. *Neisseria meningitidis*
8. *Pseudomonas aeruginosa*
9. *Shigella dysenteriae*
10. *Bacillus anthracis*
11. *Clostridium difficile*
12. *Clostridium tetani*
13. *Enterococcus faecalis*
14. *Escherichia coli*

15. *Haemophilus influenzae*
