# Supplementary material for: Assessment of the efficacy of ChatGPT responses to bacterial species-specific questions in microbiology
Source: Access Microbiol. 2026 Aug 4;8(8):001137.v5. doi: 10.1099/acmi.0.001137.v5 (PMC13449012; doi:10.1099/acmi.0.001137.v5)
Supplement: Supplementary file 2 [file acmi-8-01137-s003.pdf]

<u>Supplementary file 2- Supplementary table 1</u>

<u>1). Maximum scoring responses</u>

<table>
<tr><th>ChatGPT model and proficiency</th><th>Figure and Text</th></tr>
<tr><td rowspan="2">ChatGPT 3.5 model – High proficiency</td><td>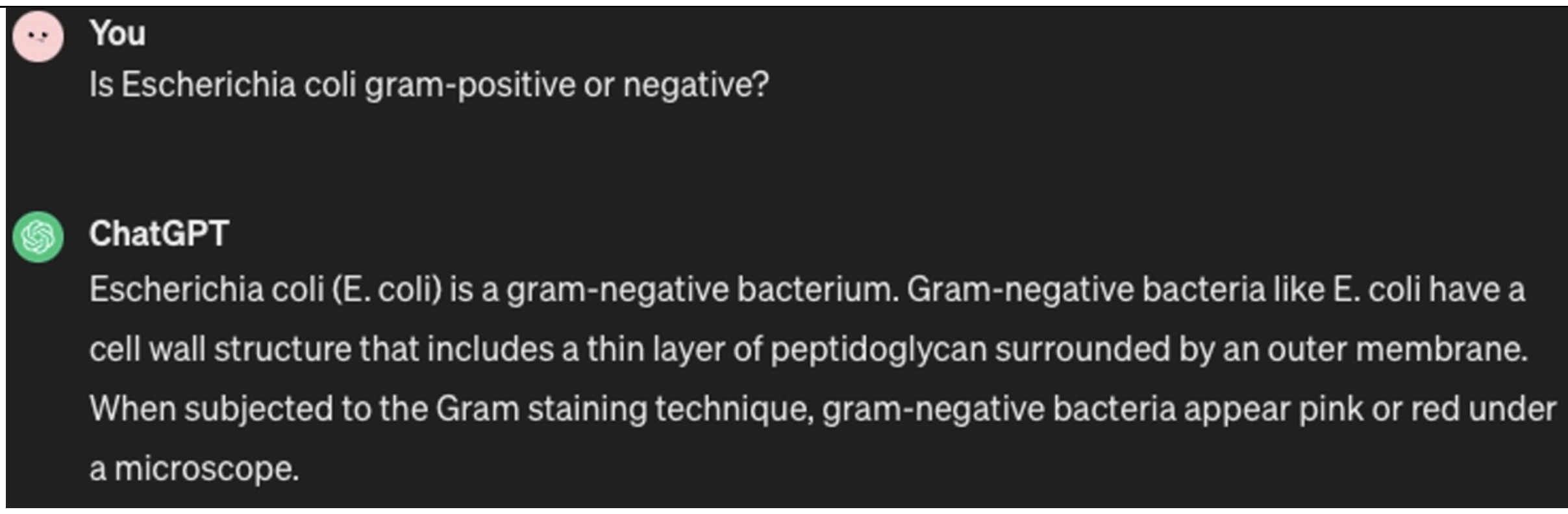
You
Is Escherichia coli gram-positive or negative?
ChatGPT
Escherichia coli (E. coli) is a gram-negative bacterium. Gram-negative bacteria like E. coli have a cell wall structure that includes a thin layer of peptidoglycan surrounded by an outer membrane. When subjected to the Gram staining technique, gram-negative bacteria appear pink or red under a microscope.</td></tr>
<tr><td>Is Escherichia coli gram positive or negative?

Escherichia coli (E. coli) is a gram-negative bacterium. Gram-negative bacteria like E. coli have a cell wall structure that includes a thin layer of peptidoglycan surrounded by an outer membrane.

When subjected to the Gram staining technique, gram-negative bacteria appear pink or red under a microscope.</td></tr>
</table>

**ChatGPT 4.1 mini model – High proficiency**	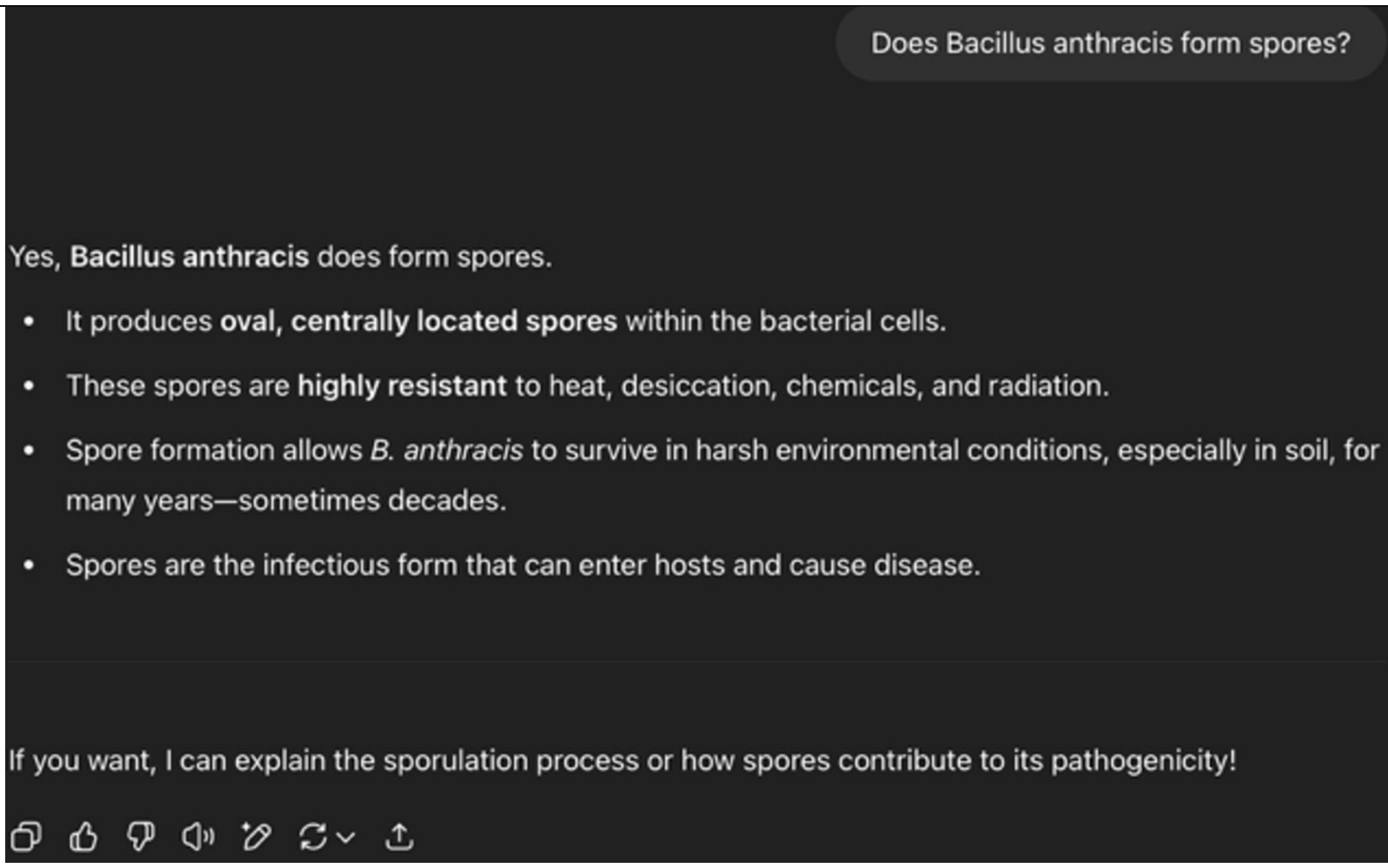<br>Does Bacillus anthracis form spores?<br>Yes, **Bacillus anthracis** does form spores.<br>• It produces **oval, centrally located spores** within the bacterial cells.<br>• These spores are **highly resistant** to heat, desiccation, chemicals, and radiation.<br>• Spore formation allows *B. anthracis* to survive in harsh environmental conditions, especially in soil, for many years—sometimes decades.<br>• Spores are the infectious form that can enter hosts and cause disease.<br>If you want, I can explain the sporulation process or how spores contribute to its pathogenicity!
	**Does Bacillus anthracis form spores?**<br><br>Yes, Bacillus anthracis does form spores.<br>• It produces oval, centrally located spores within the bacterial cells.<br>• These spores are highly resistant to heat, desiccation, chemicals, and radiation.<br>• Spore formation allows B. anthracis to survive in harsh environmental conditions, especially in soil, for many years-sometimes decades. Spores are the infectious form that can enter hosts and cause disease.<br><br>If you want, I can explain the sporulation process or how spores contribute to its pathogenicity!

2). Minimum scoring responses

ChatGPT model and proficiency	Figure and Text
ChatGPT 3.5 model – Low proficiency	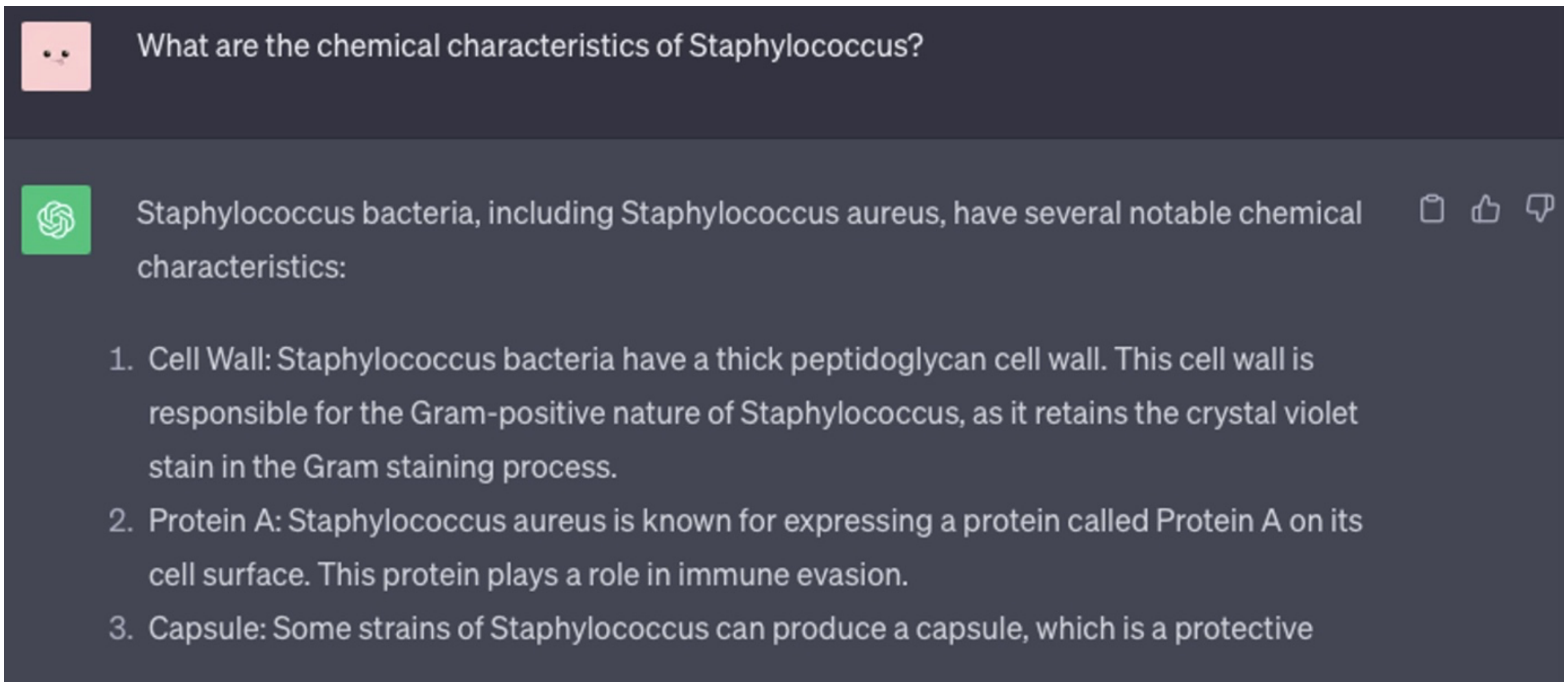<br>What are the chemical characteristics of Staphylococcus?<br>Staphylococcus bacteria, including Staphylococcus aureus, have several notable chemical characteristics:<br>1. Cell Wall: Staphylococcus bacteria have a thick peptidoglycan cell wall. This cell wall is responsible for the Gram-positive nature of Staphylococcus, as it retains the crystal violet stain in the Gram staining process.<br>2. Protein A: Staphylococcus aureus is known for expressing a protein called Protein A on its cell surface. This protein plays a role in immune evasion.<br>3. Capsule: Some strains of Staphylococcus can produce a capsule, which is a protective
	**What are the chemical characteristics of Staphylococcus?**<br><br>Staphylococcus bacteria, including Staphylococcus aureus, have several notable chemical characteristics:<br><br>• Cell Wall: Staphylococcus bacteria have a thick peptidoglycan cell wall. This cell wall is responsible for the Gram-positive nature of Staphylococcus, as it retains the crystal violet stain in the Gram staining process.

	• Protein A: Staphylococcus aureus is known for expressing a protein called Protein A on its cell surface. This protein plays a role in immune evasion.<br>• Capsule: Some strains of Staphylococcus can produce a capsule, which is a protective
**ChatGPT 3.5 model – Moderate proficiency**	What are the identified diseases caused by C.tetani?<br><br>**ChatGPT**<br>Clostridium tetani is the bacterium responsible for causing tetanus, a serious and potentially fatal disease characterized by muscle stiffness and spasms. Tetanus occurs when the spores of Clostridium tetani enter deep tissue wounds, where they germinate and produce tetanus toxin. The toxin travels through the nervous system, causing the symptoms of tetanus, which can include jaw stiffness (lockjaw), difficulty swallowing, muscle stiffness and spasms, fever, and sweating.<br><br>Other diseases caused by Clostridium species include:<br><br>1. **Clostridium difficile infection (CDI):** This infection occurs when the normal balance of bacteria in the colon is disrupted, often due to antibiotic use. C. difficile bacteria produce toxins that can cause diarrhea and, in severe cases, life-threatening colon inflammation.<br>2. **Gas gangrene (Clostridial myonecrosis):** This condition is caused by Clostridium perfringens, Clostridium septicum, Clostridium novyi, Clostridium histolyticum, and other Clostridium species. It involves the rapid destruction of muscle tissue and can lead to systemic infection and shock.<br>3. **Botulism:** Although not caused by Clostridium tetani, botulism is caused by Clostridium botulinum, another member of the Clostridium genus. Botulism is characterized by muscle paralysis and can be caused by consuming food contaminated with botulinum toxin.<br>4. **Necrotizing enterocolitis (NEC):** Clostridium perfringens is associated with NEC, a serious condition that affects premature infants and causes inflammation and necrosis in the intestines.

What are the identified diseases caused by C. tetani?

Clostridium tetani is the bacterium responsible for causing tetanus, a serious and potentially fatal disease characterized by muscle stiffness and spasms. Tetanus occurs when the spores of Clostridium tetani enter deep tissue wounds, where they germinate and produce tetanus toxin.

The toxin travels through the nervous system, causing the symptoms of tetanus, which can include jaw stiffness (lockjaw), difficulty swallowing, muscle stiffness and spasms, fever, and sweating.

Other diseases caused by Clostridium species include:

1. Clostridium difficile infection (CDI): This infection occurs when the normal balance of bacteria in the colon is disrupted, often due to antibiotic use. C. difficile bacteria produce toxins that can cause diarrhea and, in severe cases, life-threatening colon inflammation.
2. Gas gangrene (Clostridial myonecrosis: This condition is caused by Clostridium perfringens, Clostridium septicum, Clostridium novyi, Clostridium histolyticum, and other Clostridium species. It involves the rapid destruction of muscle tissue and can lead to systemic infection and shock.
3. Botulism: Although not caused by Clostridium tetani, botulism is caused by Clostridium botulinum, another member of the Clostridium genus. Botulism is characterized by muscle paralysis and can be caused by consuming food contaminated with botulinum toxin.
4. Necrotizing enterocolitis (NEC): Clostridium perfringens is associated with NEC. a serious condition that affects premature infants and causes inflammation and necrosis in the intestines.

<table>
<tr>
<td rowspan="2">ChatGPT 4.1 mini model – Low proficiency</td>
<td>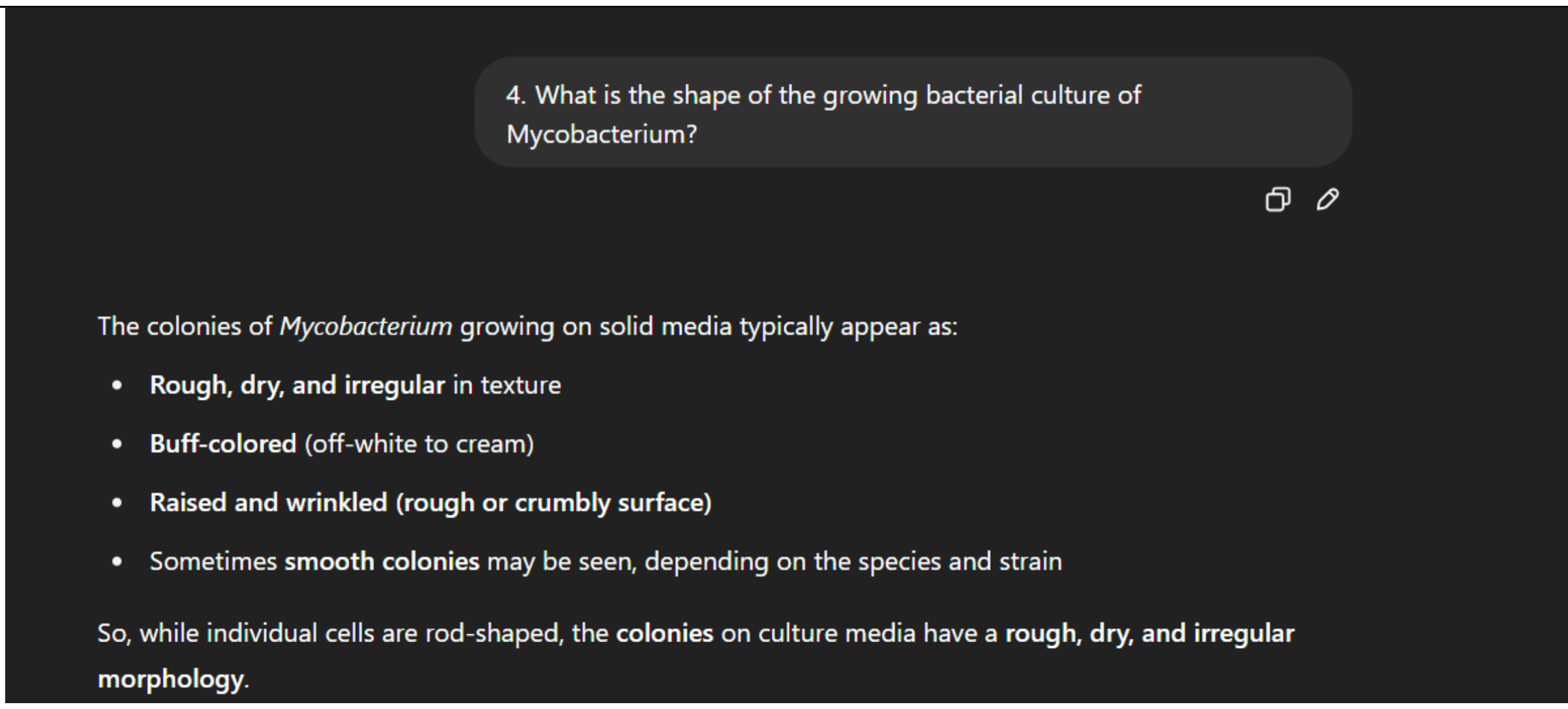
4. What is the shape of the growing bacterial culture of Mycobacterium?

The colonies of Mycobacterium growing on solid media typically appear as:
• Rough, dry, and irregular in texture
• Buff-colored (off-white to cream)
• Raised and wrinkled (rough or crumbly surface)
• Sometimes smooth colonies may be seen, depending on the species and strain

So, while individual cells are rod-shaped, the colonies on culture media have a rough, dry, and irregular morphology.</td>
</tr>
<tr>
<td>What is the shape of the growing bacterial culture of Mycobacterium?

The colonies of Mycobacterium growing on solid media typically appear as:
• Rough, dry, and irregular in texture
• Buff-colored (off-white to cream)
• Raised and wrinkled (rough or crumbly surface)
• Sometimes smooth colonies may be seen, depending on the species and strain

So, while individual cells are rod-shaped, the colonies on culture media have a rough, dry, and irregular morphology.</td>
</tr>
</table>

<table>
<tr>
<td rowspan="2">ChatGPT 4.1 mini model – Moderate proficiency</td>
<td>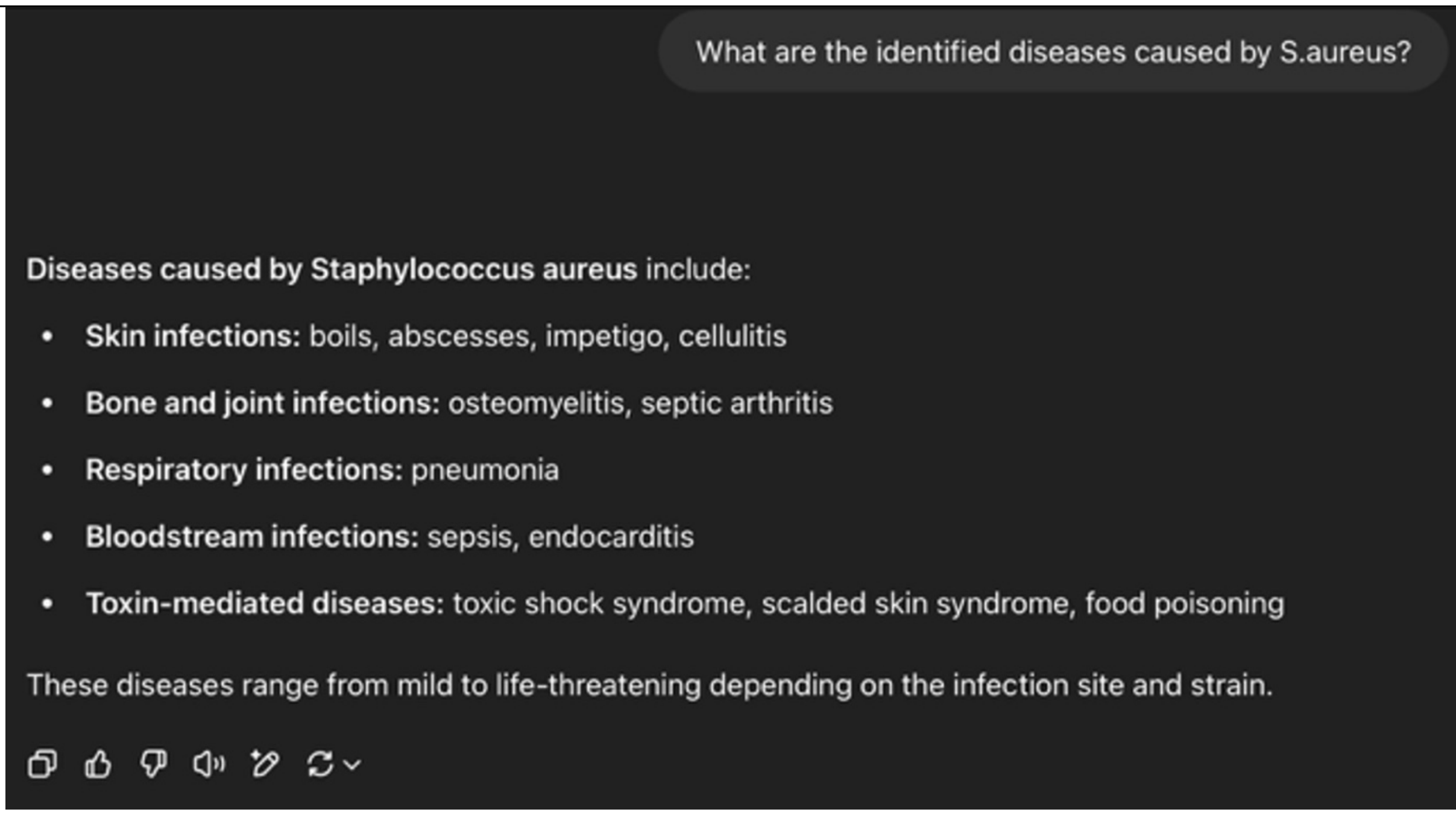
</td>
</tr>
<tr>
<td>What are the identified diseases caused by S. aureus?

Diseases caused by Staphylococcus aureus include:
• Skin infections: boils, abscesses, impetigo, cellulitis
• Bone and joint infections: osteomyelitis, septic arthritis
• Respiratory infections: pneumonia
• Bloodstream infections: sepsis, endocarditis
• Toxin-mediated diseases: toxic shock syndrome, scalded skin syndrome, food poisoning

These diseases range from mild to life-threatening depending on the infection site and strain.</td>
</tr>
</table>
